# *In Silico* and *in Vitro*-Guided Identification of Inhibitors of Alkylquinolone-Dependent Quorum Sensing in *Pseudomonas aeruginosa*

**DOI:** 10.3390/molecules23020257

**Published:** 2018-01-28

**Authors:** Fadi Soukarieh, Eduard Vico Oton, Jean-Frédéric Dubern, Janice Gomes, Nigel Halliday, Maria de Pilar Crespo, Jonathan Ramírez-Prada, Braulio Insuasty, Rodrigo Abonia, Jairo Quiroga, Stephan Heeb, Paul Williams, Michael J. Stocks, Miguel Cámara

**Affiliations:** 1 School of Life Sciences, Centre for Biomolecular Sciences, University of Nottingham, Nottingham NG7 2RD, UK; fadi.soukarieh@nottingham.ac.uk (F.S.); Eduard.VicoOton@nottingham.ac.uk (E.V.O.); jean.dubern@nottingham.ac.uk (J.-F.D.); jg1409@gmail.com (J.G.); Nigel.Halliday@nottingham.ac.uk (N.H.); Stephan.Heeb@nottingham.ac.uk (S.H.); paul.williams@nottingham.ac.uk (P.W.); 2 Department of Microbiology, Universidad del Valle and Departamento of Biomedical Sciences, Universidad Santiago de Cali, Cali AA 760035, Colombia; maria.crespo.ortiz@correounivalle.edu.co; 3 Department of Chemistry, Universidad del Valle, Cali AA 25360, Colombia; jon_ramirez94@hotmail.com (J.R.-P.); braulio.insuasty@correounivalle.edu.co (B.I.); rodrigo.abonia@correounivalle.edu.co (R.A.); jairo.quiroga@correounivalle.edu.co (J.Q.); 4 School of Pharmacy, Centre for Biomolecular Sciences, University of Nottingham, Nottingham NG7 2RD, UK; Michael.Stocks@nottingham.ac.uk

**Keywords:** *Pseudomonas aeruginosa*, PqsR, MvfR, *Pseudomonas* quinolone signal (PQS), alkylquinolone, quorum sensing inhibition.

## Abstract

*Pseudomonas aeruginosa* is a major opportunistic pathogen in cystic fibrosis, wound and nosocomial infections, posing a serious burden to public health, due to its antibiotic resistance. The *P. aeruginosa* Pseudomonas Quinolone System (*pqs*) quorum sensing system, driven by the activation of the transcriptional regulator, PqsR (MvfR) by alkylquinolone (AQ) signal molecules, is a key player in the regulation of virulence and a potential target for the development of novel antibacterial agents. In this study, we performed *in silico* docking analysis, coupled with screening using a *P. aeruginosa* mCTX::P*_pqsA_*-*lux* chromosomal promoter fusion, to identify a series of new PqsR antagonists. The hit compounds inhibited pyocyanin and alkylquinolone signal molecule production in *P. aeruginosa* PAO1-L and PA14 strains. The inhibitor **Ia**, which showed the highest activity in PA14, reduced biofilm formation in PAO1-L and PA14, increasing their sensitivity to tobramycin. Furthermore, the hepatic and plasma stabilities for these compounds were determined in both rat and human *in vitro* microsomal assays, to gain a further understanding of their therapeutic potential. This work has uncovered a new class of *P. aeruginosa* PqsR antagonists with potential for hit to lead optimisation in the search for quorum sensing inhibitors for future anti-infective drug discovery programs.

## 1. Introduction

Antimicrobial resistance has emerged as a global threat to public health, driven by complex resistance mechanisms, a lack of new antibiotics and the misuse of clinically approved antibacterial agents [1,2]. In recent years, a novel approach to combat antimicrobial resistance has begun to attract attention, based on disarming bacterial virulence through the disruption of bacterial quorum sensing (QS)-mediated communication systems. QS employs diffusible signals, sometimes called autoinducers (AIs), to control bacterial community behaviour by co-ordinating gene expression at the population level, to promote pathogen survival and dissemination [3]. There are several approaches to target QS from a drug discovery point of view and these include inhibition of the biosynthesis of AIs, blocking the interaction of these signals with their receptors or through direct interference with the signals, using enzymes or antibodies [4,5,6]. Among the organisms most clinically resistant to antibiotics is *Pseudomonas aeruginosa*, an opportunistic gram-negative pathogen and leading cause of diverse nosocomial infections, mainly in immunocompromised patients and individuals with cystic fibrosis [7,8]. *P. aeruginosa* infections are commonly difficult to eradicate with conventional antibiotic therapy, particularly when established as biofilms. 

*P.aeruginosa* possesses three integrated quorum sensing circuits, known as *las*, *rhl* and the Pseudomonas Quinolone System (*pqs*). The *las* and *rhl* systems are reliant on *N*-acylhomoserine lactones (AHLs), whereas the *pqs* system utilises alkylquinolone (AQ) derived signal molecules [9]. The latter is regulated by the interaction between the transcriptional regulator, PqsR (MvfR), and 2-heptyl-3-hydroxy-4(1*H*)-quinolone (PQS) or 2-heptyl-4-hydroxyquinoline (HHQ), which in turn induce the transcription of the *pqsABCDEphnAB* operon, leading to the production of more AQs and virulence factors, including pyocyanin as well as enhancing biofilm maturation [10,11]. It has been well documented that interfering with PqsR activity disrupts biofilm development and increases sensitivity to antibiotics, further validating its potential as a therapeutic target [12]. While the *las* and *rhl* systems have been studied extensively, with many medicinal chemistry approaches described for developing inhibitors that block binding of AHLs to their cognate receptors [13,14,15,16,17], the *pqs* system, as a therapeutic target, has received less attention. Amongst the limited number of PqsR inhibitors are quinazolinone, quinolinone, benzamide-benzimidazole and hydroxybenzamide derived molecules [18,19,20,21,22]. In this study, we employed an *in silico* virtual screening method to search for novel PqsR inhibitors in a library of quinoline-based molecules. Following that, the compounds were evaluated for their inhibitory activity, using a suite of *in vitro* assays, to reveal a new generation of PqsR inhibitors with low micromolar potency. The antagonists were then profiled for their metabolic stability in both humans and rats (plasma and liver microsomes), to gain a better understanding of their therapeutic potential.

## 2. Results

### 2.1. In Silico Virtual Screening for PqsR Antagonists

**Figure 1:**
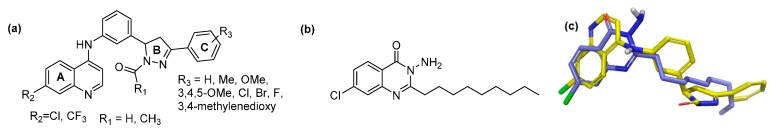
Structures of PqsR antagonists. (**a**) Chemical structures of quinolone-based compound library; (**b**) Chemical structure of PqsR inhibitor 3-NH_2_-7-Cl-C9-QZN (QZN); (**c**) Overlay of a quinolone derivative (yellow) and QZN inhibitor (blue).

We employed a virtual screening approach, to identify new inhibitors of PqsR from a library of quinolone-derived molecules. The library was based on compounds, originally reported by Ramírez-Prada et al. [23] as antiprotozoal agents, as they share a 7-chloro-4-aminoquinoline core (Figure 1a) which closely resembles the native *P. aeruginosa* AQ co-inducer head group. The study was performed using the crystal structure of the PqsR ligand binding domain, complexed with the quinazolinone inhibitor, 3-NH_2_-7Cl-C9-QZN (abbreviated as QZN), reported by Ilangovan et al. [18] (Figure 1b). We speculated that 7-chloro-4-aminoquinoline could form a suitable isostere for the head group in the QZN antagonist, while the hydrophobic aromatic extension attached to the amino group could replace the C9 alkyl chain in QZN (Figure 1c). Hence the Glide module of the Schrödinger Suite was used to examine a total of 31 structures exploiting the extra-precision docking function [24].

**Figure 2:**
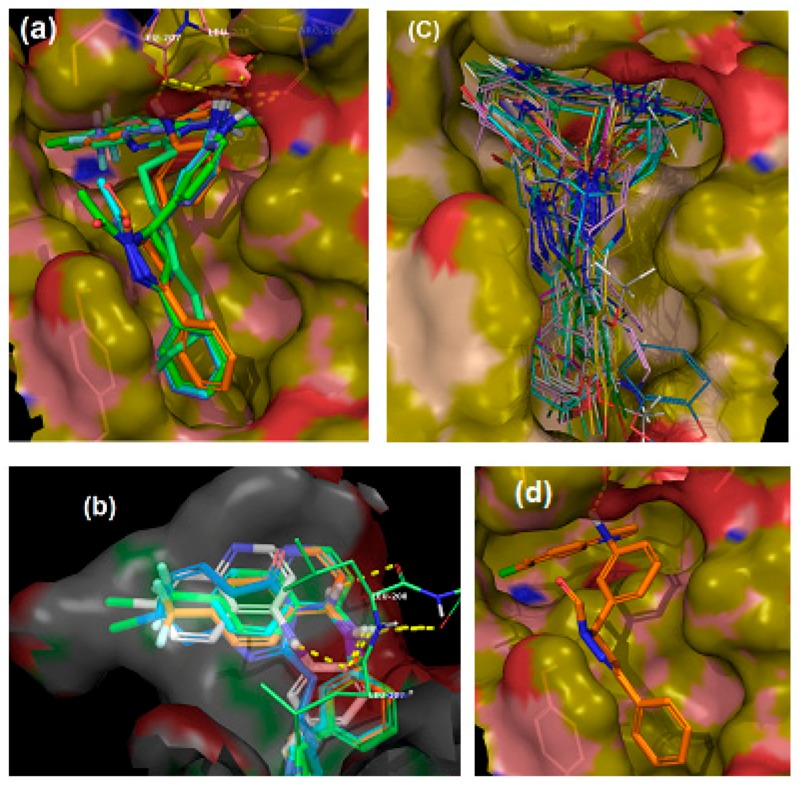
*In silico* binding of putative inhibitors to the PqsR ligand binding domain. (**a**) Overlay of the highest scored compounds in the PqsR ligand binding domain, compared to QZN (solid green); (**b**) Overlay of quinoline core in the inner pocket of PqsR; (**c**) Overlay of the entire library of compounds in the PqsR binding domain; (**d**) Binding position for compound **Ia**.

The ligand binding pocket in PqsR consists of an inner pocket (B pocket) which accommodates the quinoline head group and an elongated outer ‘A’ pocket, rich with hydrophobic residues that interact with the aliphatic chain of the co-inducers. The docking study confirmed that the favorable binding position for the quinoline library imitates the QZN inhibitor binding mode, where the 4-amino-quinoline core resides in the A pocket, while the aromatic tail extends to occupy the B pocket (Figure 2c). Moreover, the chlorine atom at the 7-position of the quinoline adopts the same orientation as the chlorine in the QZN and occupies the same small pocket formed by Thr265. The docking score, represented as XP GScore (Table 1), suggested that the best potential candidates are those with an unsubstituted ring C, while having a formyl or acetyl group on the pyrazoline ring B had no significant effect. The best fit compounds were **Ia**, **Ii**, **IIa** and **IIi** (Table 1).

Interestingly, chlorine substitution at the 7-position of the quinoline ring resulted in a slightly higher GLIDE score, compared to the corresponding trifluoromethane-substituted compounds (e.g., compare **Ia** and **Ii**). The docking positions for these ligands indicated the presence of π-π interactions between ring C and Tyr258 as well as electrostatic contacts between the quinolone 4-amino substituent and the side chains of Leu207, Leu208 and Arg209 (Figure 2a,b,d). 

### 2.2. Whole Cell Biosensor Reporter Screening for PqsR Inhibitors

**Figure 3:**
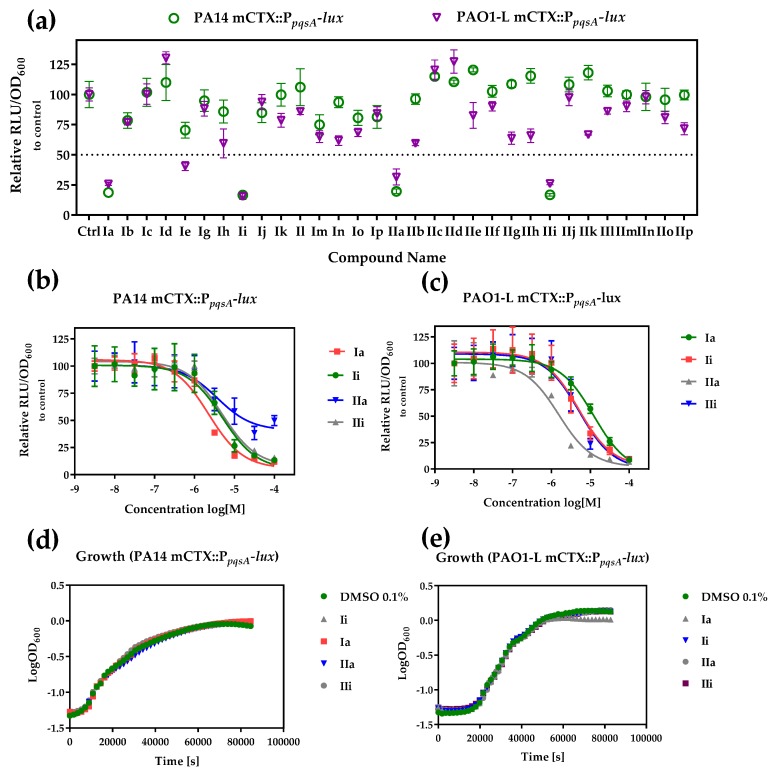
Whole bacterial cell-based *P. aeruginosa* compound screen for both PqsR and growth inhibition (**a**) Activity against PA14 (green) and PAO1-L (purple) P*_pqsA_*-*lux* reporter strains at 10 µM test compound; (**b**) and (**c**) Dose-response curves for active compounds against PA14 (**b**) and PAO1-L (**c**); (**d**) and (**e**) growth curves for PA14 (**d**) and PAO1-L (**e**) in the presence of compound or DMSO control.

To determine the ability of the quinolines to inhibit PqsR functionality, the *P. aeruginosa* biosensor strains, PAO1-L and PA14, incorporating a chromosomal mCTX::P*_pqsA_*-*lux* transcriptional fusion, were used. These report on the activation of the *pqsA* promoter, regulated by PqsR [25]. PAO1-L and PA14 were chosen because they are genetically amenable and belong to the two major *P. aeruginosa* genomic groups [26]. The quinolone compounds were incubated with the corresponding strain, and luminescence and optical density readings were recorded to monitor the effect of the quinolines on PqsR activity and bacterial growth, respectively. The compounds were screened at 10 µM concentration, and the activity was compared to a control of DMSO (0.1%) (Figure 3a). To be considered a hit, a threshold of at least 50% inhibition of the test compound, relative to the negative control had to be reached, without affecting bacterial growth. The library showed variable activity towards the reporters (Table 1, Figure 3a) and a total of four inhibitors (**Ia**, **Ii**, **IIa** and **Iii**) were identified with strong PqsR inhibitory activity for both *P. aeruginosa* strains, without interfering with bacterial growth (Figure 3a,d,e). Concentration-response experiments were then carried out to determine the IC_50_ values (Figure 3b,c), which are summarised in Table 1.

**Ii** and **IIi** had the same PqsR inhibitory activity against both strains (IC_50_ 5 µM), while compound **Ia** was the most active inhibitor of PA14 (IC_50_ 2.2 µM), but exhibited only modest activity against PAO1-L. In contrast, **IIa** was the most active PqsR antagonist for PAO1-L (IC_50_ 1.5 µM), compared with PA14 (IC_50_ of 3.7 µM).

### 2.3. Impact of PqsR Antagonists on Pyocyanin Production

**Figure 4:**
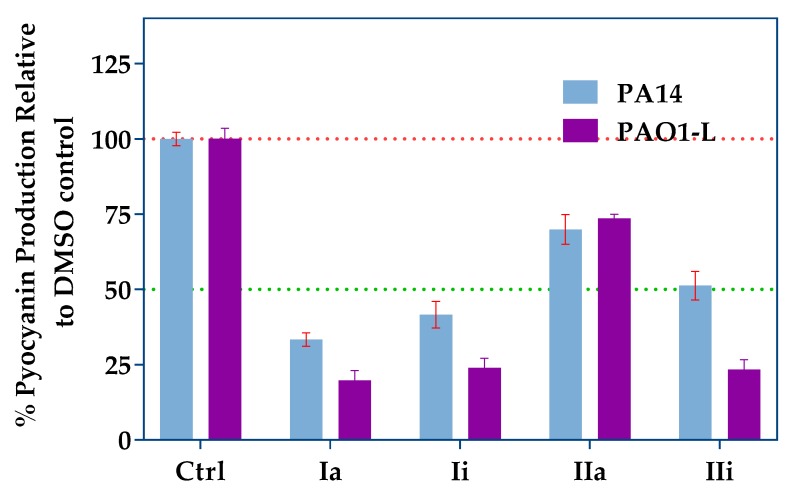
Pyocyanin production assay using the top hits at concentrations equal to three times their IC_50_s. Blue bars represent PA14 and purple bars represent PAO1-L. Error bars represent standard deviation of n = 3 biological replicates.

Pyocyanin is a blue redox-active toxin, produced by *P. aeruginosa* and strongly-controlled by PqsR *via* the regulation of *pqsE* expression [11,27,28]. Pyocyanin is essential for the pathogenicity of *P. aeruginosa* in lung infections [29]. The effect of the PqsR inhibitors on pyocyanin production by PA14 and PAO1-L strains, when grown in the presence or absence of the inhibitors, was investigated (Figure 4). All the compounds tested demonstrated significant inhibition when used at a concentration equivalent to three-fold their IC_50_ values. Compound **Ia** had the strongest effect on pyocyanin production in both strains, followed by **Ii** and **IIi**, while **IIa** had the weakest effect, with just over 25% reduction, despite its low micromolar potency in the reporter assay. 

### 2.4. Impact of Hit Compounds on Alkylquinolone Production

**Figure 5:**
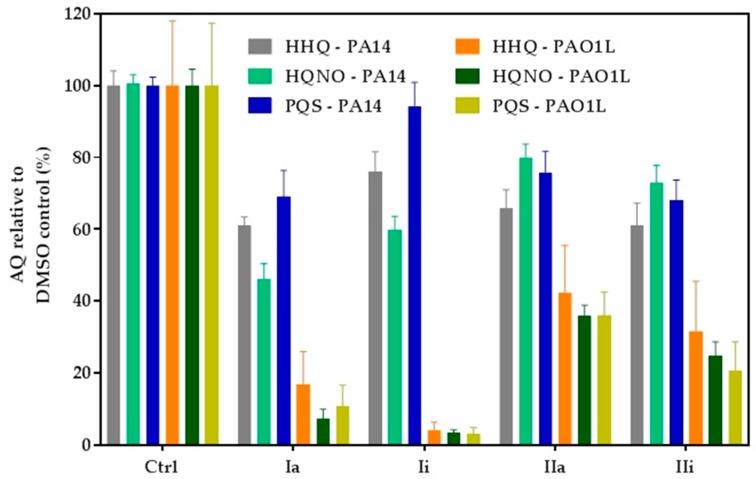
Inhibition of alkylquinolone (AQ) production. PAO1-L and PA14 were treated with the corresponding PqsR inhibitors at 3× IC_50_ for 16 h and culture supernatants extracted for LC-MS/MS analysis. Each experiment was performed using three biological and three technical replicates. The bars show percentage levels in relation to a non-treated DMSO control.

*P. aeruginosa* produces a broad range of alkylquinolones, *via* PqsABCDE, some of which (e.g., PQS and HHQ) act as quorum sensing signal molecules, while others (e.g., 2-heptyl-4-hydroxyquinoline *N*-oxide; HQNO) do not [11,30]. HQNO, can protect *Staphylococcus aureus* from vancomycin [31] and contributes to the environmental competitiveness of *P. aeruginosa*, through its activity as a potent inhibitor of the cytochrome *bc*_1_ complex [32]. AQ biosynthesis is tightly-regulated by PqsR, hence inhibition of this transcriptional regulator is reflected by a reduction in AQ production that can be used as a readout for PqsR inactivation. LC-MS/MS was used to quantify the major AQs HHQ, PQS and HQNO after incubation of the *P. aeruginosa* strains with the hit compounds, for 16 h. The assay was performed using both PAO1-L and PA14 strains which were treated with PqsR antagonists at concentrations three-fold higher than their IC_50_ values. The results obtained depended on the inhibitor and strain tested (Figure 5). Compound **Ii** showed the greatest inhibition of AQ production in PAO1-L; however, this was not observed for PA14. Furthermore, **IIa** had only a moderate effect, whereas **Ia** maintained a strongly-inhibited AQ production for both strains, in agreement with the pyocyanin data (compare Figure 4 and Figure 5). 

### 2.5. Effect of PqsR Antagonists on Biofilms

One of the clinical challenges of *P. aeruginosa* infections is the difficulty associated with the effective treatment of biofilms due to their high resistance to antibiotics. The *pqs* system has been shown to regulate a number of genes required for biofilm development [27] and hence, inactivation of this system through antagonism of PqsR is a promising approach, as it can sensitize biofilms to conventional antibiotics [33]. The effect of **Ia,** with or without tobramycin, on PA14 and PAO1-L biofilms grown on glass coverslips, was determined. **Ia** was the compound of choice, as it demonstrated relatively strong inhibitory activity in each of the assays used in this study. The experiment was performed using green fluorescent labelled (GFP)-labelled PAO1-L and PA14 strains, grown under four different conditions: (i) no treatment (control); (ii) compound **Ia** at 8 µM in PA14 and 34 µM in PAO1-L (concentrations equal to 3× IC_50_); (iii) tobramycin at 100 µg/mL; (iv) **Ia** and tobramycin, at the same concentrations as (ii) and (iii). 

**Figure 6:**
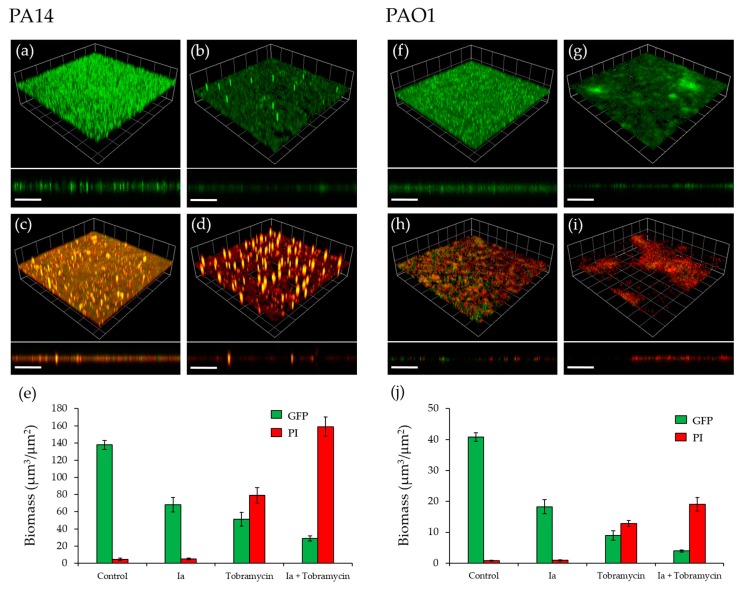
Effect of **Ia** on *P. aeruginosa* biofilms. (**a**) Untreated GFP-labelled PA14 biofilm; (**b**) GFP- labelled PA14 biofilm grown with **Ia** at 8 µM; (**c**) GFP-labelled PA14 biofilm, treated with 100 µg/mL tobramycin for 4 h after 16 h of growth; (**d**) GFP-labelled PA14 biofilm grown with 8 µM **Ia** and treated with 100 µg/mL tobramycin for 4 h after 16 h of growth; (**e**) Biomass quantitation of PA14 biofilms; (**f**) Untreated GFP-labelled PAO1-L biofilm; (**g**) GFP-labelled PAO1-L biofilm grown with **Ia** at 34 µM; (**h**) GFP-labelled PAO1-L biofilm, treated with 100 µg/mL tobramycin for 4 h after 16 h of growth; (**i**) GFP-labelled PAO1-L biofilm grown with 34 µM **Ia** and treated with 100 µg/mL tobramycin for 4 h after 16 h of incubation; (**j**) Biomass quantitation of PAO1-L biofilms. Dead cells and extracellular DNA were stained with propidium iodide (PI). Three-dimensional (3D) sections and cross sections are shown. Scale bars represent 100 µm.

Figure 6 shows that exposure to **Ia** had a substantial effect on biofilm development for both PAO1-L and PA14, reducing biomass with little impact on cell viability, as measured using propidium iodide (red bars) (Figure 6a,b,f,g,e,j). Treatment with tobramycin significantly reduced biofilm biomass, increasing the proportion of dead bacterial cells substantially (Figure 6c,h,e,j). Most importantly, the combined treatments had a synergistic effect with near complete biofilm eradication (Figure 6d,i,e,j). These data further validate the *pqs* system in *Pseudomonas* as a target that can aid the effectiveness of antibiotic treatment for biofilm-centred infections.

### 2.6. Determination of the Plasma and Hepatic Stability of Selected Quinolone Inhibitors

To gain a greater understanding of the potential of the quinolone inhibitors to progress towards a hit to lead optimization process, *in vitro* plasma and hepatic stability assays were performed. The results obtained (Table 2) show that each of the selected compounds possesses a high degree of stability in rat plasma and to a less extent in human plasma, where the half-lives varied, with the most stable compound being **IIa** (Table 2). Microsomal stability usually represents Phase 1 metabolism of a drug, although it does not incorporate factors such as protein binding, which can lead to enhanced *in vivo* stability. The majority of the compounds tested demonstrated moderate to high intrinsic clearance with relatively short half-lives of <30 min in both human and rat microsomes. Inhibitors **Ia** and **Iii** showed the greatest plasma stability. 

## 3. Discussion

Antimicrobial resistance has become a global challenge with very slow progress in the development of novel therapeutics [2]. As a multi-antibiotic resistant Gram-negative pathogen, *P. aeruginosa* constitutes a major clinical threat [34,35]. There is a growing body of literature describing several approaches to attenuate bacterial virulence through interference with the expression or activity of virulence factors [36]. Despite some reports on the emergence of resistance to these agents, these approaches are becoming more popular for treating bacterial infections, as they pose less selective pressure on the bacterial pathogen [37,38]. Disruption of QS can provide an alternative approach to conventional antibacterial therapy, as it targets bacterial virulence, rather than bacterial viability [4,39,40,41]. The *pqs* system plays a critical role in regulating virulence and biofilm development, through the transcriptional regulator, PqsR [42]. In this study, we employed a molecular modelling approach using the Schrödinger Suite [24] to assess a series of 4-amino-quinoline-based compounds with three sites for amenable structural modifications. To our knowledge, this is the first report describing the use of this *in silico* approach to search for PqsR inhibitors. Our docking results highlighted four compounds with the potential to bind to PqsR, adopting similar conformations to the reported QZN inhibitor [15]. The interactions within the PqsR ligand-binding site and **Ia**, **Ii**, **Ia**, **Ii** were mainly dominated by a hydrophobic π-π interaction with Tyr258 and electrostatic interactions between the 4-amino and the side chains of Leu207, Leu208 and Arg209. Moreover, the chlorine and trifluoromethane groups at the 7-position adopted similar orientations towards Thr265, in agreement with the previously-reported binding orientation for QZN [18]. It is noteworthy that substitution at the para-position was not tolerated and resulted in a substantial reduction in the GLIDE docking score, as this may cause a change of conformation to the phenyl ring, affecting the π-π interaction with Tyr258. To confirm that these results can be translated to *P. aeruginosa* cells, we used biosensor reporter strains, in which a chromosomal transcriptional fusion of P*_pqsA_*-*lux* was introduced. This responds to endogenous AQ production by emitting light. In the presence of an antagonist, bioluminescence is reduced, providing a simple read-out [25]. Compounds active in this reporter assay may either be inhibitors of PqsR or of AQ biosynthetic enzymes. The results obtained with the quinolines confirmed that compounds (**Ia**, **Ii**, **Ia**, **Ii**) are active in both *P. aeruginosa* strains tested at low micromolar concentrations. In addition, there were some other hits with selective activity against one of the strains. These results highlight the importance of including multiple strains to validate the activity of an inhibitor, as its potency may vary in a strain-dependent manner. Interestingly, all reported PqsR inhibitors to date were examined using a single *P. aeruginosa* strain, highlighting the need for these compounds to be tested in different strains before further optimisation is carried out [19,22,43,44]. In fact, it is crucial that anti-*Pseudomonas* agents undergo a rigid assessment of their activity in representative strains and fresh clinical isolates before they can be considered candidates, as this would greatly reduce the chances of expensive failure at the clinical stage, due to lack of potency [26]. The reasons for such differences are not clear, as yet, but may be attributable to different cell envelope permeabilities, active efflux pumps or a secondary mode of action at a different target [45]. In the present study, only compounds that showed consistent potency in both PA14 and PAO1-L were further assessed using phenotypic analysis. 

A pyocyanin quantitation assay upon treatment with the compounds revealed that **Ia** was the most effective at reducing phenazine production, followed by **Ii**. Surprisingly, **IIa** reduced pyocyanin modestly, despite being the most potent inhibitor in the bioreporter assay. In agreement with our findings for pyocyanin, **Ia** was the most potent inhibitor of AQ production, followed by **IIa**, the effect of which was substantial for PAO1-L. The remaining inhibitors showed only modest, variable effects on AQ production. Collectively, the inhibitory activity of the compounds, alongside the *in silico* modelling, is consistent with PqsR as their target. However, the P*_pqsA_*-*lux* reporter assay used does not distinguish between inhibitors of PqsR and AQ biosynthetic enzymes, and further work will be required to confirm that PqsR is their direct target [19,22,43,44]. Based on the results obtained, **Ia** was evaluated for anti-biofilm activity. **Ia** disrupted biofilm development and reduced biomass, without affecting bacterial viability. Furthermore, **Ia** sensitized the biofilm to tobramycin, as reflected by an improved potency in killing both PAO1-L and PA14 biofilms, a finding that supports previous reports that inhibition of *pqs* signalling disrupts biofilm integrity and its sensitivity to antibiotic treatment [33,46].

Before progressing a hit to lead optimisation process, it is important to determine the stability of the most active hits in plasma and hepatic microsome assays. The latter experiments are essential for predicting the metabolic stability of a putative drug and aid medicinal chemistry decisions [47,48]. The stability study showed that these inhibitors present a reasonable level of plasma stability; however, the intrinsic clearance values in hepatic microsomes were elevated and their half-lives were relatively short (less than 30 min). Thus, further chemical refinement to improve hepatic stability should be considered if these molecules are to be pursued as leads. Nevertheless, we have introduced a new class of low micromolar putative PqsR inhibitors that constitute a starting point for further medicinal chemistry studies aiming at optimising their potency and physicochemical properties, to generate clinically useful therapeutic agents particularly as adjuvants for antibiotics in the context of *P. aeruginosa* biofilm-centred infections.

## 4. Materials and Methods 

### 4.1. Molecular Docking

#### 4.1.1. Preparation of Protein and Receptor Grid Generation

The X-ray crystal structure of PqsR ligand binding domain in complex with the QZN inhibitor (PDB ID: 4JVI) was used as a protein template. The protein was prepared using the protein preparation wizard (Small-Molecule Drug Discovery Suite 2017-4, Schrödinger, LLC, New York, NY, USA), where hydrogen atoms were added, water molecules were removed and the correct bond order was assigned to the amino acid residues. Afterwards, a receptor grid generation was performed, based on defined residues around the ligand binding sites: (Ala102, Pro129, Ile149, Thr166, Ala 168, Val 170, Ile186, Ile189, Gln194, Ser196, Leu196, Leu197, Leu207, Leu208, Pro210, Val211, Trp234, Gly235, Pro238, Ser255). The inner grid box was set to 10 Å, while the outer box was 20 Å.

#### 4.1.2. Ligand Preparation

The chemical structures of the inhibitors were sketched using ChemDraw (Version 16.0.1.4, PerkinElmer informatics) via an SDF file. LigPrep module (Small-Molecule Drug Discovery Suite 2017-4, Schrödinger, LLC, New York, NY, USA) was then used for final preparation of ligands into their lowest energy 3D conformations. The partial atomic charges were assigned to the molecular structures, using the 2005 implementation of the OPLS-AA force field. These energy-minimized structures were used for Glide) docking.

#### 4.1.3. Molecular Docking

The “Extra Precision” (XP) mode of Glide docking (Small-Molecule Drug Discovery Suite 2017-4, Schrödinger, LLC, New York, NY, USA) was used to perform all docking calculations, using the OPLS-AA 2005 force field. The scale factor of 1.0 for van der Waals radii was applied to atoms of protein with absolute partial charges of less than or equal to 0.25. The number of position per ligand was set to five, after energy minimization. The best docked structures were chosen using an XP Glide Score (XP Gscore) function as well as visual observations.

### 4.2. Bacterial strains and growth conditions

The *P. aeruginosa* strains and plasmids used in this study are shown in Table 3. Bacteria were grown in lysogeny broth (LB) at 37 °C, unless stated otherwise. Where required, tetracycline (Tc) was added to the media at 125 µg/mL, to select for recombinants. Synthetic alkylquinolones were added at the concentrations indicated.

### 4.3. Biosensor Reporter Assay

Strains PA14 mCTX::P*_pqsA_*-*lux* and PAO1-L mCTX::P*_pqsA_*-*lux* were constructed using plasmid mini-CTX::P*_pqsA_*-*lux*, as previously described [50], and the assay was performed according to a published protocol [52]. For screening, the compounds were tested at a concentration of 10 µM, which was prepared from a 10 mM stock, in DMSO.

### 4.4. Pyocyanin Quantification

The experiment was performed following a published protocol with minor modifications [53]. *P. aeruginosa* strains were cultured into 5 mL fresh medium overnight. Compounds were assayed at 3× IC_50_s, for 16 h, at 37 °C (Kuhner LT W Shaker, Adolf Kühner AG, Basel, Switzerland). Cells were centrifuged at 10,000 RCF for 10 min (Allegra 64R centrifuge, Beckman Coulter, High Wycombe, UK) and the supernatant was transferred to 15 mL falcon tubes with a HSW 10 mL Soft-Ject Syringe and a 0.22 μM Sartorius syringe-driven filter (Fisher Brand, Loughborough, UK). Pyocyanin pigment was extracted into chloroform by mixing 7.5 mL of supernatant with 4.5 mL of chloroform. Pyocyanin was further extracted into 1.5 mL of 0.2 M HCL, which gave a pink/red solution, and the absorbance was measured at 520 nm.

### 4.5. LCMS-MS Alkyl Quinoline Quantification

For each test sample, 100 µL of sterile filtered supernatant (the same preparation as for pyocyanin) was spiked with 10 µL of an internal standard solution (10 µM d4-PQS in MeOH), and diluted with water, to a total volume of 500 µL. Samples were then extracted three times with an 0.5 mL aliquot of ethyl acetate, vortex mixing the aqueous/organic mix for 2 min, then removing the organic phase once the layers had successfully partitioned. For each sample, the combined organic extracts were dried under vacuum and re-dissolved in 100 µL MeOH prior to analysis. For the LC-MS/MS analysis of supernatant extracts, the chromatography was achieved using a Shimadzu series 10AD VP LC system (Columbia, MD, USA). The LC column, maintained at 40 °C, was a Phenomenex Gemini C18 (3.0 µm, 100 × 3.0 mm) (Macclesfield, Cheshire, UK) with an appropriate guard column. Mobile phase A was 0.1% (*v*/*v*) formic acid in water containing 2 mM 2-picolinic acid, and mobile phase B 0.1% (*v*/*v*) formic acid in methanol. The flow rate throughout the chromatographic separation was 450 µL/min. After an injection of a 2 µL/sample, a binary gradient, beginning initially at 30% B, increased linearly to 99% B over 5 min. The composition remained at 99% B for 3 min, decreased to 30% B over 1 min, and stayed at this composition for 4 min, to allow for column equilibration. The MS system used for analyte detection was an Applied Biosystems Qtrap 4000 hybrid triple-quadrupole linear ion trap mass spectrometer (Foster City, CA, USA), equipped with an electrospray ionisation (ESI) interface. Instrument control, data collection and analysis were conducted using Analyst software (Foster City, CA, USA). The MS analysis was achieved with positive electrospray (+ES) multiple reaction monitoring (MRM) screening of the LC eluent for specific AQ analytes. Where chromatographic peaks for HHQ, HQNO and PQS were detected, a peak area was determined, and analyte peak area/internal standard peak area calculated.

### 4.6. Biofilms

Biofilms were cultivated on borosilicate glass coverslips in petri dishes. *P. aeruginosa* strains, PAO1-L and PA14, were labelled by transformation with plasmid pMMG, which constitutively expresses GFP from the P*_tac_* promoter [51]. Labelled strains were grown at 37 °C, for 16 h, in 2 mL RPMI-1640 (Lonza, Slough, UK), supplemented with 20 mM D-glucose (Sigma–Aldrich, Dorset, UK) and 2 µM FeCl_3_ (Sigma–Aldrich, Dorset, UK). Cultures were diluted 1:100 in fresh medium and allowed to grow for a further 4 h, or until an OD_600_ of 0.5 was reached. The mid-logarithmic cultures were diluted to an OD_600_ of 0.01 in 25 mL RPMI, supplemented with glucose and FeCl_3_, and inoculated into petri dishes containing UV sterilised borosilicate glass coverslips (22 × 22 mm, thickness no1) (VWR, Lutterworth, UK). Bacterial cells were seeded at 37 °C under static conditions for 1.5 h, and compound **Ia** was added to the culture at a concentration of 34 µM for *P. aeruginosa* PAO1-L and 8 µM for PA14 before dishes were moved to a shaker at 60 rpm and 37 °C for 15 h to form mature biofilms. Tobramycin and propidium iodide were added to the 15 h-old cultures at concentrations of 100 µg/mL and 2 µM, respectively, followed by further incubation for 4 h. Coverslips were examined under a Laser Scanning Fluorescent Microscope (LSM2, Zeiss, Oberkochen, Germany). Biofilms were visualised using *egfp* mode at an excitation wavelength of 488nm. Imaging was carried out using Zen 2011 imaging software (Zeiss, Oberkochen, Germany). A total of 5 Z-stacked images were collected per coverslip. Sampling was conducted at random from the central portion of each coverslip. Biomass was calculated using Image J (NIH, Bethesda, MD, USA) and Comstat 2.1. Software package (www.comstat.dk, lyngby, Denmark) [54].

### 4.7. Determination of Plasma Stablity

Dilutions of a 10 mM test compound DMSO stock solution were prepared so that the final DMSO concentration was 1% *v*/*v* and the final test compound concentration tested was 1 µM. Following the addition of the test compound to plasma, the samples were pre-incubated for 10 min prior to the start of the incubation by the addition of the test compound. The test compound was incubated (n = 2; 1 µM final concentration) at 37 °C with plasma for up to 2 h. Aliquots were sampled at several time points and mixed with acetonitrile (containing internal standard) to terminate the reaction and precipitate the proteins. All the samples were mixed, centrifuged and the supernatants analysed by UPLC-MSMS. Parent disappearance and half-life (t 1/2) values were determined from the slope of the parent depletion curve. Control compounds (imidapril, enalapril, tenofovir and/or propantheline), were respectively included.

### 4.8. Determination of Hepatic Stability

Dilutions of a 1 mM test compound DMSO stock solution were prepared in buffer (typically 0.01 M phosphate buffered saline (pH 7.4) so that the final DMSO concentration was 0.1% *v*/*v* and the final test compound concentration tested was 1 µM. Following addition of protein (0.5 mg/mL final) and NADPH, the samples were pre-incubated for 10 min prior to the start of the incubation by the addition of test compound. The test compounds were then incubated (n = 2; 1 µM final concentration) at 37 °C with tissue microsomes. Aliquots were sampled at several time points and mixed with acetonitrile (containing internal standard) to terminate the reaction and precipitate the proteins. All the samples were mixed, centrifuged and the supernatants analysed by UPLC-MS/MS. The intrinsic clearance (Clint) and half-life (t 1/2) values were determined from the slope of the parent depletion curve. Control compounds for low, moderate and high intrinsic clearance, respectively, for both human and rat microsomes, were included.

### 4.9. Data Analysis and Figure Preparation

Sigmoidal dose-response curves and the representation of all data were prepared using GraphPad Prism 7.

## Figures and Tables

**Table 1:** Summary of docking scores and activity data for the library.

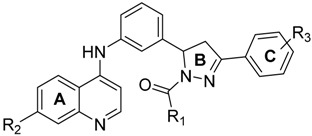								
**ID**	**R_1_**	**R_2_**	**R_3_**	**Glide** **XP Score**	PA14 Remaining Activity % *	PAO1-L Remaining Activity % *	**IC_50_ PA14** µM *	**IC_50_ PAO1-L** µM *
**Ia**	-H	-Cl	4-H	−9.856	18.7 ± 1.98	25.8 ± 1.17	2.3 ± 0.51	12.4 ± 1.79
**Ib**	-H	-Cl	4-Me	−5.179	78.4 ± 6.51	77.0 ± 2.25		
**Ic**	-H	-Cl	4-OMe	−6.984	101.8 ± 11.67	100.3 ± 8.46		
**Id**	-H	-Cl	3,4,5-OMe	−7.265	109.9 ± 14.98	130.3 ± 5.04		
**Ie**	-H	-Cl	4-F	−5.24	70.4 ± 6.53	40.6 ± 3.71		
**Ig**	-H	-Cl	4-Br	−6.34	94.8 ± 8.91	88.1 ± 6.05		
**Ih**	-H	-Cl	3,4-Methy-lenedioxy	−4.056	85.8 ± 9.42	49.9 ± 12.01		
**Ii**	-H	-CF_3_	4-H	−8.087	16.6 ± 1.3	15.6 ± 2.53	5.0 ± 0.82	5.1 ± 0.32
**Ij**	-H	-CF_3_	4-Me	−6.868	84.9 ± 8.17	93.7 ± 6.15		
**Ik**	-H	-CF_3_	4-OMe	−4.867	99.7 ± 9.37	78.6 ± 5.75		
**Il**	-H	-CF_3_	3,4,5-OMe	−2.219	106 ± 15.27	85.8 ± 2.39		
**Im**	-H	-CF_3_	4-F	−7.118	74.9 ± 8.28	65.1 ± 4.89	-	
**In**	-H	-CF_3_	4-Cl	−6.044	93.6 ± 4.45	61.8 ± 3.97		
**Io**	-H	-CF_3_	4-Br	−6.390	80.6 ± 6.26	68.5 ± 3.43		
**Ip**	-H	-CF_3_	3,4-Methy-lenedioxy	−6.099	81.4 ± 9.36	84.3 ± 5.48		
**IIa**	-Me	-Cl	4-H	−8.918	19.7 ± 1.9	31.6 ± 6.60	4.0 ± 1.62	1.6 ± 0.29
**IIb**	-Me	-Cl	4-Me	−5.0855	96.2 ± 4.34	59.5 ± 1.86		
**IIc**	-Me	-Cl	4-OMe	−6.58033	114.9 ± 2.13	120.3 ± 8.28		
**IId**	-Me	-Cl	3,4,5-OMe	−6.5015	110.4 ± 1.6	127.3 ± 9.69		
**IIe**	-Me	-Cl	4-F	−7.1105	120.4 ± 1.59	82.6 ± 22.73		
**IIf**	-Me	-Cl	4-Cl	−7.732	102.6 ± 4.91	90.2 ± 3.84		
**IIg**	-Me	-Cl	4-Br	−4.9555	108.6 ± 2.79	63.7 ± 5.01		
**IIh**	-Me	-Cl	3,4-Methy-lenedioxy	−4.13	115.4 ± 6.02	65.8 ± 5.63		
**IIi**	-Me	-CF_3_	4-H	−8.5165	16.8 ± 1.01	26.1 ± 0.99	4.9 ± 0.30	5.14 ± 0.60
**IIj**	-Me	-CF_3_	4-Me	−5.371	108.2 ± 6.05	97.3 ± 6.46		
**IIk**	-Me	-CF_3_	4-OMe	−6.799	118.1 ± 5.97	66.7 ± 0.42		
**IIl**	-Me	-CF_3_	3,4,5-OMe	−7.006	102.9 ± 4.84	86.1 ± 2.39		
**IIm**	-Me	-CF_3_	4-F	−3.578	99.9 ± 3.17	90.2 ± 4.40		
**IIn**	-Me	-CF_3_	4-Cl	−5.533	98.1 ± 11.38	97.8 ± 5.52		
**IIo**	-Me	-CF_3_	4-Br	−7.5535	95.6 ± 9.58	80.8 ± 5.06		
**IIp**	-Me	-CF_3_	3,4-Methylenedioxy	−6.8975	99.6 ± 4.09	71.6 ± 5.07		

* Values are reported as Mean ± SD of n = 3 replicates.

**Table 2:** Summary of plasma and microsomal stability of PqsR inhibitors.

	Plasma Stability		Microsomal Stability			
	***Rat***	***Human***	***Rat***		***Human***	
**Compound ID**	**t_1/2_** **(min)**	**t _1/2_** **(min)**	Clint * (µL/min/mg)	**t_1/2_ (min)**	**Clint (µL/min/mg)**	**t_1/2_ (min)**
**Ia**	>240	132.2	122.5	11.5	60.3	23.3
**Ii**	>240	69.1	54.1	25.7	80.7	17.2
**IIa**	>240	73.8	87.8	15.8	73.0	19.0
**IIi**	>240	157.1	65.7	21.1	72.9	19.0

* Clint: intrinsic clearance.

**Table 3:** Bacterial strains and plasmids used in this study.

Strain or Plasmid	Relevant Characteristics	Reference or Origin
*P. aeruginosa*		
PAO1-L	Wild type PAO1, Lausanne subline.	B. Holloway, *via* D. Haas
PAO1-L mCTX::P*_pqsA_*-*lux*	PAO1-L with chromosomal mini-CTX::P*_pqsA_*-*lux* insertion; Tc^R^	This study
PA14	Wild type UCBPP-PA14	[49]
PA14 mCTX::P*_pqsA_*-*lux*	PA14 with chromosomal mini-CTX::P*_pqsA_*-*lux* insertion; Tc^R^	This study
Plasmids		
mini-CTX::P*_pqsA_*-*lux*	R6K-based mini-CTX suicide plasmid for the chromosomal insertion of a P*_pqsA_*-*lux* transcriptional reporter fusion; Tc^R^	[50]
pMMG	pME6032∆*lacI* constitutively expressing GFP from the P*_tac_* promoter	[51]
